# The effect of a brief, unplanned treatment delay on neovascular age-related macular degeneration patients: a retrospective cohort study

**DOI:** 10.1038/s41598-023-29819-y

**Published:** 2023-02-23

**Authors:** Jason Adam Zehden, Arko Ghosh, Srinath Soundararajan, Tamy Harumy Moraes Tsujimoto, Huijun Jiang, Feng-Chang Lin, Tyler Blahnik, David Fleischman, Alice Yang Zhang

**Affiliations:** 1 Department of Ophthalmology, University of North Carolina (UNC), 2226 Nelson Hwy Suite 220, Chapel Hill, NC 27517 USA; 2 Department of Ophthalmology, University of Arizona, Tucson, AZ USA; 3 School of Medicine, University of North Carolina (UNC), Chapel Hill, NC USA; 4 Department of Biostatistics, University of North Carolina (UNC), Chapel Hill, NC USA

**Keywords:** Macular degeneration, Retinal diseases

## Abstract

Non-compliance to intravitreal anti-vascular endothelial growth factor (anti-VEGF) therapy can result in increased disease activity in neovascular age-related macular degeneration (nAMD). Our study aims to determine effects of unplanned delay in anti-VEGF injection treatment for nAMD. This retrospective observational study included patients with delays in receiving intravitreal injections for nAMD treatment from March to May 2020 by at least 21 days. Baseline demographic and clinical characteristics, visual acuity (VA), central macular thickness (CMT) measured on optical coherence tomography (OCT), and duration of delayed treatment were analyzed for 3 time points, the pre-delay visit (v1) and post-delay visits (v2 and v3). Data were compared to age-matched controls treated for nAMD in 2019 without delay. Demographic characteristics were compared using two-sample t-tests for continuous variables and Pearson’s chi-square tests for categorical variables. For the two primary outcomes of interest, VA and CMT, means and standard deviations were reported for each combination of group and time. Each outcome was modeled using a linear mixed model with the group, time and group-time interaction as fixed effects. A total of 69 patients (99 eyes) in the treatment delay group and 44 patients (69 eyes) in the control group were identified. Statistically significant differences between control and delayed groups were detected for VA (difference in mean logMAR = 0.16; 95% CI 0.06, 0.27; p = 0.002) and CMT (difference in mean CMT = 29; 95% CI 12, 47; p = 0.001) at v2. No differences were detected for v1 and v3 time points for both outcomes. An unplanned delay in intravitreal injection treatment for nAMD resulted in an increase in CMT and worsening of VA compared to controls observed at v2. At v3, CMT and VA recovered to near v1 levels. This study demonstrates that a one-time, brief interruption in treatment for nAMD results in reversible, temporary worsening.

## Introduction

Patients with neovascular age-related macular degeneration (nAMD), an advanced form of age-related macular degeneration (AMD), may develop irreversible vision loss due to retinal damage caused by hemorrhage and exudation. The first-line treatment for nAMD is intravitreal anti-vascular endothelial growth factor (VEGF) injections and non-compliance to regular anti-VEGF treatment can result in increased disease activity and decreased visual acuity^1,2^. The World Health Organization declared a global pandemic due to Severe Acute Respiratory Syndrome Coronavirus 2 (SARS-CoV-2), the virus responsible for Coronavirus disease 19 (COVID-19), on March 11, 2020, and subsequently, the American Academy of Ophthalmology (AAO) stated on March 18, 2020, that ophthalmologists should cease providing any treatment unless it was deemed urgent or emergent^3,4^. The American Society of Retinal Specialists (ASRS) then released a report describing intravitreal injections as essential to preventing permanent vision loss^5^. The ASRS stated that the risk of vision loss from missing injections must be weighed against the risk of infection exposure and that patients receiving injections were also most at risk for morbidity and mortality from COVID-19^5^. Due to the risks imposed by the COVID-19 pandemic, many patients receiving intravitreal anti-VEGF injections had unplanned delays in receiving their injections. The simultaneous interruption of therapy for a large group of patients in the same timeframe created a study population for examining the effects of a brief, unplanned intravitreal anti-VEGF injection treatment delay.

This study aimed to examine the short and long-term effects on logMAR best-corrected visual acuity (VA) and central macular thickness (CMT) in patients with nAMD who had at least a 3-week delay receiving intravitreal anti-VEGF treatment.

## Materials and methods

This was a retrospective observational study conducted in the Department of Ophthalmology at the University of North Carolina at Chapel Hill. It included patients with nAMD who were scheduled to receive an intravitreal injection (bevacizumab, aflibercept, or ranibizumab) for the treatment of nAMD from March to May 2020 but were delayed in the context of the COVID-19 pandemic (treatment delay group). This study was conducted in accordance with the Declaration of Helsinki. Ethical approval was granted by the Institutional Review Board at the University of North Carolina at Chapel Hill.

The study included patients that had at least a 21-day delay in receiving their scheduled anti-VEGF intravitreal injection and had at least 16 weeks of follow-up after their first appointment following the delay. Data were also collected from age and sex-matched controls who were treated for nAMD in 2019 without treatment delays (control group). Patients were excluded from the study if their treatment delay was less than 21 days, they had less than 16 weeks of follow-up after their first post-delay visit, or if they were receiving intravitreal injections for an indication other than nAMD. Patients were excluded from the control group if there were treatment delays during the study period. Data were collected from medical records of patients in the study, including age, sex, race, intravitreal anti-VEGF agent used, the CMT, and the Snellen VA which was converted to logMAR VA. Information was collected from three visits for each group. The three visits for the treatment delay group were the pre-delay visit (v1), the first post-delay visit (v2), and a 2nd post-delay visit at least 16 weeks following visit 2 (v3). V3 was a follow up visit that was closest to and at least 16 weeks after v2, but v3 was not required to be the immediate next visit after v2. The follow up time of 16 weeks post-v2 was chosen because it was estimated to be a sufficient amount of time since v2 to detect if patients’ VA and CMT would demonstrate any recovery. In the control group, v1 was the baseline visit, v2 was the first regularly scheduled treatment visit, and v3 was 6 months following v2. The primary outcomes measured were the comparison of the CMT measured on optical coherence tomography (OCT) and logMAR VA between the pre-delay visit (v1) and two post-delay visits (v2 and v3). All initial and follow up CMT measurements for each patient were obtained with the same spectral domain OCT machine (Heidelberg engineering).

Demographic characteristics of participants were reported with frequencies, percentages, means, and standard deviations when appropriate. The two groups were compared in those characteristics using two-sample t-tests for continuous variables and Pearson’s chi-square tests for categorical variables. Paired t-tests were used in the intra-group comparison of the anti-VEGF injection interval at v1 versus v3. For the two primary outcomes of interest, logMAR VA and CMT, means and standard deviations were reported for each combination of group and time, with 95% confidence intervals plotted in the figure. Each outcome was modeled using a linear mixed model with the group, time and group-time interaction as fixed effects. A random intercept was added into the model to account for the dependence between the eyes of the same patient. Statistical analyses were performed in R version 4.0.2 (R Core Team, 2020). Complete-case analysis was considered when there is missing data, with a p-value < 0.05 determining statistical significance.

### Ethics approval and consent to participate

The study was approved and the need for informed consent was waived by the Institutional Review Board at the University of North Carolina at Chapel Hill (IRB No. 20-1286). The need for informed consent was waived due to the retrospective nature by the Institutional Review Board at the University of North Carolina at Chapel Hill. Nothing is specifically linked to any patients.

## Results

Ninety-nine eyes of 69 patients were included in the treatment delay group, while sixty-nine eyes of 44 patients were included in the control group. The demographic characteristics of both groups are listed in Table 1. The mean age of the treatment delay group was 84 years (± 8), 65% were female, and 88% were White. Most patients received bevacizumab (54%), while 37% received aflibercept, and 9% received ranibizumab. Prior to the treatment delay, at v1, the average anti-VEGF treatment interval was 8.27 weeks (± 2.61 weeks). The average baseline logMAR VA was 0.493 (± 0.428) and the average baseline CMT was 294 μm (± 90.4 μm). The mean number of days of treatment delay was 44 days (± 37 days). The average duration of follow-up after the first post-COVID visit was 155 days (± 43 days).

**Table 1:** Demographic characteristics.

Characteristic	Overall (N = 168)	Control group (N = 69)	Treatment delay group (N = 99)	p-value
Length of delay	44 (37)	NA (NA)	44 (37)	
Age	84 (8)	85 (7)	84 (8)	0.3
Gender				0.6
F	112 (67%)	48 (70%)	64 (65%)	
M	56 (33%)	21 (30%)	35 (35%)	
Race				0.4
Asian	7 (4.2%)	1 (1.4%)	6 (6.0%)	
Black	4 (2.4%)	2 (2.9%)	2 (2.0%)	
Hispanic	1 (0.6%)	0 (0%)	1 (1.0%)	
White	153 (91%)	66 (96%)	87 (88%)	
Unknown	3 (1.8%)	0 (0%)	3 (3.0%)	
Visit 1 injection				0.3
Bevacizumab	82 (49%)	29 (42%)	53 (54%)	
Aflibercept	69 (41%)	32 (46%)	37 (37%)	
Ranibizumab	17 (10%)	8 (12%)	9 (9.1%)	
Visit 1 injection interval in weeks	8.65 (2.91)	9.20 (3.23)	8.27 (2.61)	0.05
Number of days of follow-up since visit 1	261 (55)	258 (37)	263 (64)	0.6
Days between visit 1 to visit 2	91 (47)	66 (29)	109 (49)	< 0.001
Days of follow-up from visit 2 to visit 3	170 (46)	192 (43)	155 (43)	< 0.001
Pre-COVID VA logMAR				
Mean (SD)	0.531 (0.482)	0.585 (0.550)	0.493 (0.428)	0.25
Pre-COVID OCT CMT				
Mean (SD)	297 (109)	301 (131)	294 (90.4)	0.69
Injection interval at v3				
Mean (SD)	8.98 (2.99)	9.49 (3.19)	8.62 (2.80)	0.073
Inter-group comparison of v1 to v3 injection interval change				
Mean (SD)	0.303 (1.43)	0.235 (1.07)	0.351 (1.65)	0.59
Years receiving treatment at v1				
Mean (SD)	3.74 (2.27)	3.66 (1.66)	3.79 (2.62)	0.69
Days receiving treatment at v1				
Mean (SD)	1360 (829)	1340 (605)	1380 (958)	0.69
2 or more years of treatment at v1				
No	42 (25.0%)	12 (17.4%)	30 (30.3%)	0.085
Yes	126 (75.0%)	57 (82.6%)	69 (69.7%)	

The demographic characteristics of the control group were very similar to the treatment delay group: average age was 85 years (± 7 years), 70% were female, and 96% were White. The distribution of use of anti-VEGF agent was similar: 42% received bevacizumab, 46% received aflibercept, and 12% received ranibizumab. The average treatment interval at v1 was 9.20 weeks (± 3.23 weeks), which was statistically different but not clinically different (p = 0.05). The baseline logMAR VA was 0.585 (± 0.550) and the baseline CMT was 301 μm (± 131 μm). The average duration of follow-up was 192 days (± 43 days).

The number of days between v1 and v2 significantly differed between the treatment delay and control groups. Additionally, the number of days between v2 and v3 significantly differed between the two groups. The number of days between v1 and v3 did not significantly differ between the two groups. The only baseline characteristic that had a statistically significant difference between the two groups was the injection interval at v1. However, the injection interval at v1 of 9.20 weeks (± 3.23 weeks) in the control group compared to 8.27 weeks (± 2.61 weeks) in the treatment delay group was not clinically significant. The mean number of years that patients had been receiving intravitreal injections at v1 was 3.66 (± 1.66) in the control group and 3.79 (± 2.62) in the treatment delay group, which was not significantly different (p = 0.69). A majority of eyes in each treatment group had been receiving intravitreal injections for two or more years at v1. 57 out of 69 eyes (82.6%) in the control group had been receiving injections for two or more years at v1. 69 out of 99 eyes (69.7%) in the treatment delay group had been receiving injections for two or more years at v1. There was not a statistically significant difference in the proportion of eyes that had been receiving injections for two or more years between the two groups (p = 0.085). Other baseline characteristics did not significantly differ between the treatment-delay and control groups (Table 1).

In the treatment-delay group, the mean logMAR VA value at v1 was 0.49 (± 0.43), which worsened to 0.63 (± 0.58) at v2, and 0.59 (± 0.55) at v3. The corresponding mean CMT at v1 was 294 μm (± 90 μm), which increased to 321 μm (± 102 μm) at v2 and decreased to 291 μm (± 80 μm) at v3 (see Table 2). In the control group, the logMAR VA value at v1 was 0.58 (± 0.55), which improved to 0.56 (± 0.47) at v2, and 0.62 (± 0.59) at v3. The corresponding baseline mean CMT at v1 was 301 μm (± 131 μm), 299 μm (± 128 μm) at v2, and 291 μm (± 116 μm) at v3. The difference in the mean VA from v1 to v2 was higher in the treatment-delay group compared to the control group (0.14 vs. − 0.02; the difference is 0.16 and has 95% CI 0.06, 0.27; p = 0.002). The difference in the mean CMT from v1 to v2 was greater in the treatment-delay group compared to the control group (27 vs. − 2; the difference is 29 and has 95% CI 12, 47; p = 0.001). No statistically significant differences were found from v1 to v3 for both outcomes.

**Table 2:** Visual acuity and CMT for all visits.

Characteristic	Control group			Treatment delay group		
	Visit 1	Visit 2	Visit 3	Visit 1	Visit 2	Visit 3
logMAR	0.58 (0.55)	0.56 (0.47)	0.62 (0.59)	0.49 (0.43)	0.63 (0.58)	0.59 (0.55)
CMT	301 (131)	299 (128)	291 (116)	294 (90)	321 (102)	291 (80)

Values are presented as means with standard deviations.

**Figure 1:**
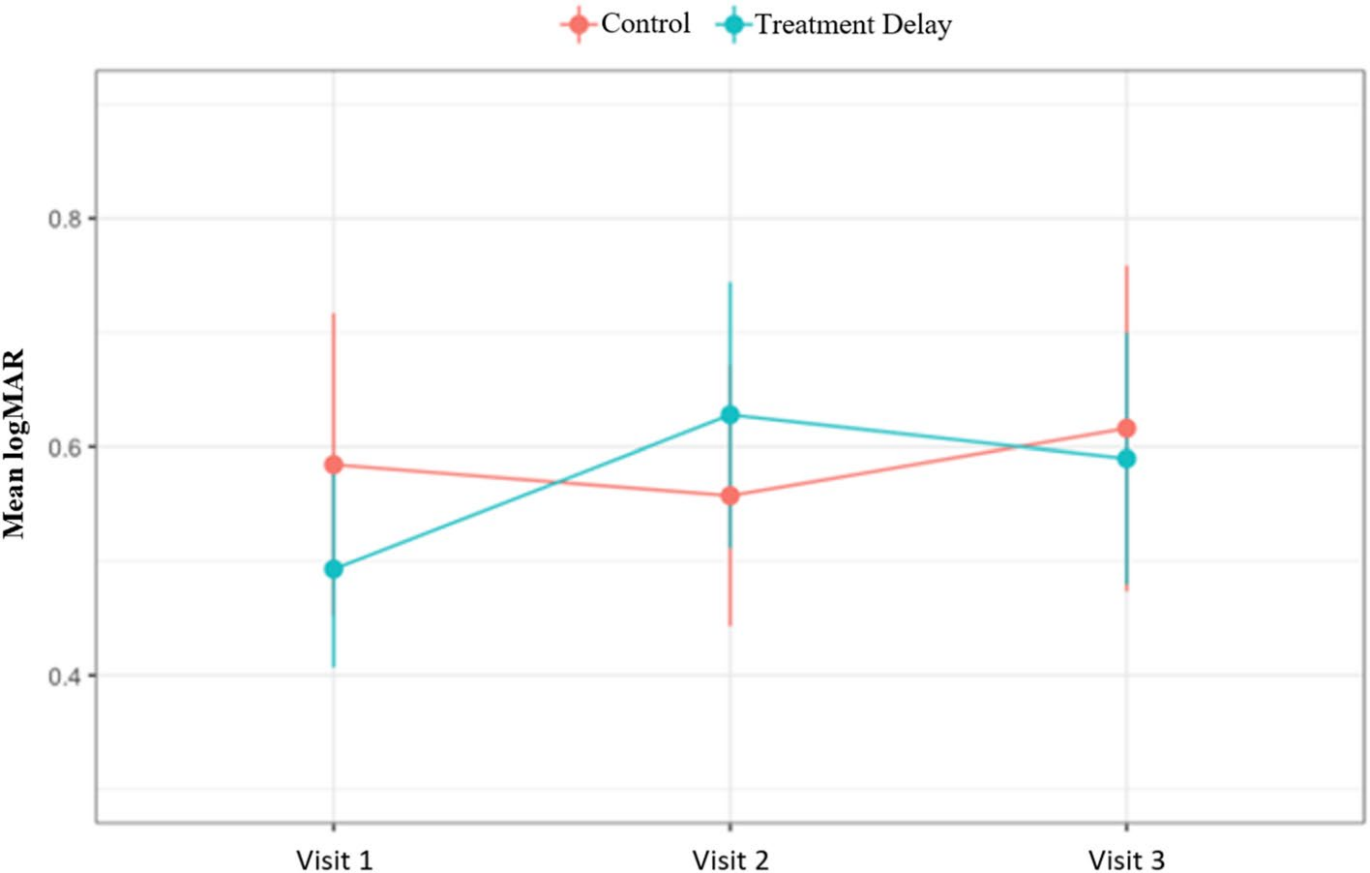
Change in visual acuity over time. Demonstrates the change in visual acuity in logMAR across each visit for both the treatment delay group and the control group.

**Figure 2:**
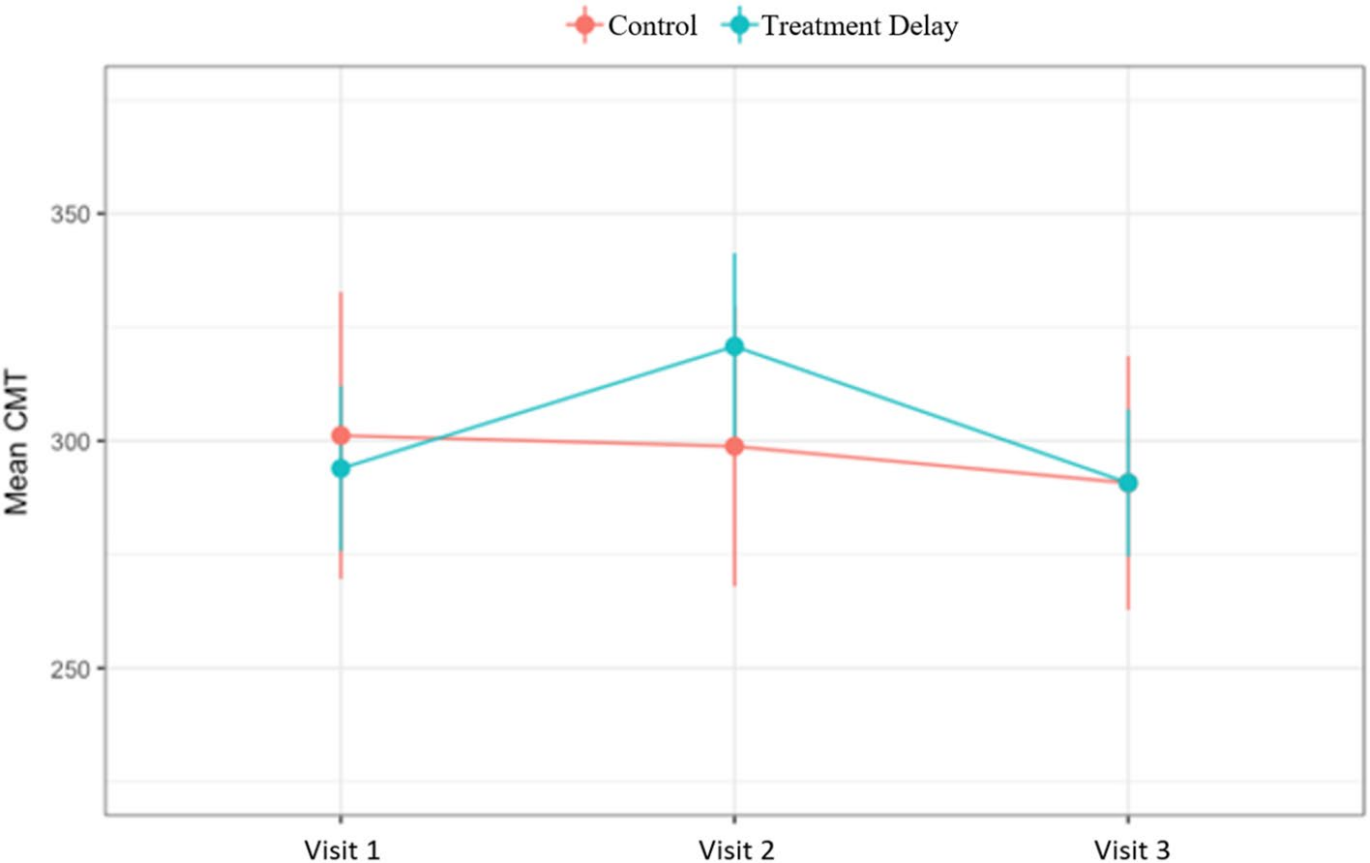
Change in CMT over time. Demonstrates the change in CMT across each visit for both the treatment delay group and the control group.

The mean logMAR VA of the treatment-delay group worsened from v1 to v2 and modestly improved from v2 to v3, while the mean VA of the control group slightly improved from v1 to v2 and worsened from v2 to v3 (see Fig. 1). The mean CMT of the treatment-delay group increased from v1 to v2 and decreased from v2 to v3, while the mean CMT of the control group gradually decreased across all three visits (see Fig. 2). The estimation result based on the linear mixed model showed a significant difference between the two groups in VA (p = 0.002) and CMT (p = 0.001) at v2. No statistically significant differences were found at v1 and v3 for both outcomes.

The anti-VEGF injection interval at v1 was not significantly different than the injection interval at v3, both when comparing v1 to v3 intra-group and inter-group. For the control group, the interval at v1 was 9.20 weeks (± 3.23 weeks) and the interval at v3 was 9.49 weeks (± 3.19 weeks), which was not a statistically significant difference (p = 0.073). For the treatment group, the interval at v1 was 8.27 weeks (± 2.61 weeks) and the interval at v3 was 8.62 weeks (± 2.80 weeks), which was a statistically significant difference (p = 0.039) but not a clinically significant difference. Additionally, the change in the injection interval from v1 to v3 in the control group, 0.235 weeks (± 1.07 weeks), compared to the change in the injection interval from v1 to v3 in the treatment delay group, 0.351 weeks (± 1.65 weeks), was not significantly different (p = 0.59). Treatment intervals ranged from 4 to 16 weeks for both groups.

Additionally, our study analyzed whether the injection interval prior to treatment delay had a significant impact on VA or CMT. A linear mixed model with injection interval at v1 as a fixed effect was used to determine if the injection interval at v1 had any interaction with VA or CMT. Using this model, we determined that the injection interval at v1 had no statistically significant effect on the change in VA or CMT.

## Discussion

It is not uncommon for patients who have been on consistent schedules for receiving intravitreal anti-VEGF injections to unexpectedly miss an injection due to illness or other unforeseen circumstances. The COVID-19 pandemic caused many patients with nAMD to have unplanned delays in receiving their intravitreal anti-VEGF injections. Both patients and ophthalmologists became concerned with the impact this would have on patients’ retinal health and vision. However, the disruption of intravitreal anti-VEGF therapy for a group of patients at the same, brief time point created a cohort to examine the effects of a short, unplanned treatment delay.

Several studies analyzed retrospective data from patients with delays in anti-VEGF treatment previously. Typically, delays in treatment were studied over a 3- to 6-month period, and not on as large a scale as the interruption caused by the COVID-19 pandemic. The three parameters of interest in this study are the effect of the treatment delay on visual acuity, effect of treatment delay on CMT on OCT, and whether visual acuity and CMT recover following a period of resumed treatment.

Soares et al. analyzed data from patients with nAMD that were lost to follow-up for at least six months but later returned for treatment^6^. This study found that VA remained significantly worse at both the 6-month return visit and the 12-month return visit^6^. Nguyen et al. found that in eyes with nAMD that had anti-VEGF injections suspended due to disease inactivity for three months, 41% had disease reactivation in the first year of treatment suspension and 79% had disease reactivation by the fifth year^7^. Furthermore, they found that patients suffered VA declines that did not recover to pre-suspension levels even after treatment was restarted^7^. Elfalah et al. found that in patients with nAMD, VA demonstrated a statistically significant deterioration when examined immediately after a delay of 2 months^8^. Sindal et al. discovered that VA of both treatment-naive and patients previously receiving anti-VEGF injections had worsening of their VAs when they first presented for treatment after a 19-week treatment delay^9^. In our study, visual acuities initially significantly worsened compared to the control group, similar to the above studies that reported worsening acuities after a period of no anti-VEGF treatment. However, after 4-months of treatments, visual acuities recovered and were not significantly different from those of control patients. This is likely due to a very short treatment delay with a mean of 44 days compared to many months.

Elfalah et al. studied change in CMT on OCT and noted no significant change when examined immediately after a delay of 2 months^8^. Navarrete et al. found that in nAMD patients with a treatment delay of a median of 21 days, 145 out 305 patients had worsened CMT^10^. Additionally, Navarrete et al. found that there was a significant correlation between length of treatment delay and increase in CMT^10^. Our study found that there was a statistically significant worsening of CMT in patients immediately after a short period of treatment delay compared to the control group which had very minimal change in CMT.

Rush et al. found that in patients that resumed anti-VEGF therapy after an average 11.8-week treatment delay and had at least 6 months of continued injections after resuming treatment, the VA and CMT both worsened compared to baseline and compared to control patients without treatment delays. After 6 months of resumed treatment, although there was a significant improvement in CMT, there was no significant improvement in VA. Neither parameter recovered to baseline levels^11^. Our study found that the initial worsening in both VA and CMT did improve after a 4-month resumed treatment period. There was a statistically significant difference in VA and CMT in the treatment delay group compared to the control group at the first follow-up. Based on the results of our study, a short delay in receiving anti-VEGF therapy did not result in long-term irreversible visual loss in patients with nAMD.

Furthermore, it is interesting to note that the anti-VEGF treatment interval remained stable post-treatment delay compared to pre-treatment delay. The majority of the patients in the treatment and control group used bevacizumab or aflibercept, and no patients required a change in anti-VEGF agent during the study period. The fact that most patients were on a stable treatment regimen, meaning they had been receiving injections for two years or more, should be taken into consideration when examining the results of our study. It is possible that eyes on stable treatment regimens for two or more years are not as affected by a brief delay in treatment compared to eyes in the loading phase or in the first year of treatment.

The main limitation of our study is that it included a small sample size for both the treatment delay group and the control group. Additionally, the small sample size resulted in a large amount of variability in the CMT and VA values. Also, since the majority of eyes in the study had been receiving anti-VEGF treatment for two years or more at v1, the results of our study cannot be generalized to eyes in the loading phase of anti-VEGF treatment or eyes with less than two years of treatment.

## Conclusions

Our study demonstrates that although there is a worsening in disease activity as measured by CMT and VA in the immediate weeks after a delay in treatment, it is encouraging that within 4 months, on average, the changes are reversible to the point that CMT and VA do not significantly differ between treatment-delayed patients and control patients. This information is useful to guide treatment decisions and expected prognosis for patients that have brief, unplanned interruptions in intravitreal anti-VEGF injections. A short delay in treatment may not negatively impact disease activity.

## Data Availability

The datasets used and/or analyzed during the current study are available from the corresponding author on reasonable request.
